# Performance and community structure dynamics of microbial electrolysis cells operated on multiple complex feedstocks

**DOI:** 10.1186/s13068-020-01803-y

**Published:** 2020-10-13

**Authors:** Scott J. Satinover, Miguel Rodriguez, Maria F. Campa, Terry C. Hazen, Abhijeet P. Borole

**Affiliations:** 1 Bredesen Center for Interdisciplinary Research and Education, The University of Tennessee, Knoxville, USA; 2 Biosciences Division, Oak Ridge National Laboratory, Oak Ridge, TN USA; 3 Civil and Environmental Engineering, The University of Tennessee, Knoxville, USA; 4 Institute for a Secure & Sustainable Environment, The University of Tennessee, Knoxville, USA; 5 Chemical and Biomolecular Engineering, The University of Tennessee, Knoxville, USA

## Abstract

**Background:**

Microbial electrolysis is a promising technology for converting aqueous wastes into hydrogen. However, substrate adaptability is an important feature, seldom documented in microbial electrolysis cells (MECs). In addition, the correlation between substrate composition and community structure has not been well established. This study used an MEC capable of producing over 10 L/L-day of hydrogen from a switchgrass-derived bio-oil aqueous phase and investigated four additional substrates, tested in sequence on a mature biofilm. The additional substrates included a red oak-derived bio-oil aqueous phase, a corn stover fermentation product, a mixture of phenol and acetate, and acetate alone.

**Results:**

The MECs fed with the corn stover fermentation product resulted in the highest performance among the complex feedstocks, producing an average current density of 7.3 ± 0.51 A/m^2^, although the acetate fed MECs outperformed complex substrates, producing 12.3 ± 0.01 A/m^2^. 16S rRNA gene sequencing showed that community structure and community diversity were not predictive of performance, and replicate community structures diverged despite identical inoculum and enrichment procedure. The trends in each replicate, however, were indicative of the influence of the substrates. *Geobacter* was the most dominant genus across most of the samples tested, but its abundance did not correlate strongly to current density. High-performance liquid chromatography (HPLC) showed that acetic acid accumulated during open circuit conditions when MECs were fed with complex feedstocks and was quickly degraded once closed circuit conditions were applied. The largest net acetic acid removal rate occurred when MECs were fed with red oak bio-oil aqueous phase, consuming 2.93 ± 0.00 g/L-day. Principal component analysis found that MEC performance metrics such as current density, hydrogen productivity, and chemical oxygen demand removal were closely correlated. Net acetic acid removal was also found to correlate with performance. However, no bacterial genus appeared to correlated to these performance metrics strongly, and the analysis suggested that less than 70% of the variance was accounted for by the two components.

**Conclusions:**

This study demonstrates the robustness of microbial communities to adapt to a range of feedstocks and conditions without relying on specific species, delivering high hydrogen productivities despite differences in community structure. The results indicate that functional adaptation may play a larger role in performance than community composition. Further investigation of the roles each microbe plays in these communities will help MECs to become integral in the 21st-century bioeconomy to produce zero-emission fuels.

## Background

Microbial electrolysis cells (MECs) are an emerging technology that may pave the way for renewable hydrogen production from wastes, gaining significant attention from researchers in the last decade. MECs have been applied to several different feedstocks, ranging from simple organic materials to complex wastes from industrial sources [1, 2]. Complex feedstocks will represent the most valuable substrates for MEC development, but MECs fed with complex feedstocks generally underperform compared to those fed with simple substrates. While most of the biological understanding is gained via MECs fed with simple substrates, the role of biological specificity and functionality in MECs fed with a complex feedstock largely remains unknown. This can be important for understanding the limitations of MECs using complex substrates and help develop new strategies for designing and operating them.

Practical deployment of MECs will benefit from using a high performing pre-developed or mature community that will adapt to new substrates, rather than growing a new community from a natural inoculum. Few studies compare the changes in microbial community or performance associated with a mature bioanode after adapting to a new substrate, and fewer studies have investigated the differences in community structure that occur when MECs are transitioned from one substrate to another. Despite the inconsistent feeding regime, the body of knowledge that compares the community and performance when fed different substrates does not have a strong consensus. Studies have shown that community structure, performance, and substrate composition may be related [3–7], while others have shown little correlation between these metrics [8–10]. Sampling differences may be playing a large role in these studies, as community structure can be dependent on where the community is sampled in the reactor. Kim et al. used a tubular MFC fed with sucrose and found 80–90% similarity in species composition across replicates, but showed that significant changes in community structure occurred between the beginning and the end of the tubular MEC [11]. Using a compact MEC fed with complex feedstocks and starting with a selectively enriched community may produce more consistent results.

Complex wastes can be composed of hundreds of individual compounds with individual degradation pathways, however, there are some key compounds of importance. Pretreatment of complex feedstocks’ often generates volatile fatty acids (VFAs) and other organic acids [12]. Feed streams may, therefore, contain these organic acids, serving as substrates for exoelectrogens. Organic acids can be produced in situ in MECs fed with complex feedstocks. Perhaps the most desirable organic acid for MECs is acetate, as it is directly converted into electrons by exoelectrogens. It may be key to high performance as an intermediate of complex feedstock degradation [13]. Other VFAs, like propionic acid, can be degraded in MECs but do so less efficiently [14, 15]. If acetate and other organic acids are critical to current production in MECs, then higher-performing MECs should accumulate and degrade these compounds at faster rates, with acetate being the most important of the organic acids. However, not all microbial compound degradation produces acetic acid as an intermediate, as some will generate other compounds that may not be substrates for exoelectrogens. Phenol and catechol can accumulate as a result of biotransformation of phenolics and furans in MECs [16, 17]. Thus, documenting the utilization of the parent compounds as well as identification of intermediates must both be done to better understand the microbial metabolism at work.

This kind of understanding is most valuable in high performing MECs, however, it requires consideration of additional issues to allow rapid development. Several operating parameters contribute to high-performing MECs [18]. Providing sufficient energy to exoelectrogens in MECs is critical, as anode poising has been shown to improve MEC performance compared to applying a whole-cell potential [19]. Mass transfer limitations from the anode to the cathode must also be minimized. Improved mass transfer can be achieved by increasing the anode liquid flow rate in flow-through systems [20]. To advance MEC technology, the simultaneous integration of effective strategies is necessary. High-performance systems can be developed by combining as many of the design and operating parameters as possible into one system [21, 22].

The goal of this study is to investigate the microbial diversity of MECs fed with complex feedstocks using MECs with previously demonstrated high performance. Thus, an MEC design that previously reported hydrogen productivities of 17.9 L/L-day using a complex substrate derived from corn stover [22] was used. The MECs were inoculated with a pre-developed community [13], grown to maturity indicated by generating the necessary current for such high performance, and investigated with five different substrates in a specific sequence and using a defined adaptation protocol. Five substrates were selected ranging widely in complexity, starting with biomass-derived complex wastes to acetate as the simplest substrate possible for exoelectrogens [23]. Electrochemical performance associated with each substrate and the MEC effluent streams’ chemical characterization was determined to track the degradation of key compounds and the accumulation of intermediates. 16S rRNA gene sequencing was used to track the MEC community structure developed using each substrate. Finally, exploratory statistical analysis was used to find similarities associated with taxonomical classification, performance metrics, and compound removal/accumulation.

## Results and discussion

### Substrate characterization

The five substrates used in the study included a pyrolysis bio-oil aqueous phase from switchgrass (BOAP) [24], a pyrolysis bio-oil aqueous phase from red oak (ROBOAP) [25], a corn stover fermentation product (CFP) [22, 26, 27], a blend of acetate and phenol (phe/ace) using equal parts grams of chemical oxygen demand (COD) per liter, and acetate. The three complex feedstocks tested contained some similar characteristics despite the differences in pretreatment used. Table 1 shows the composition of each substrate by percent COD determined by High-Performance Liquid Chromatography (HPLC) and the total COD concentration. All three complex feedstocks contained significant fractions of VFAs. The most prominent compound found in the complex feedstocks was acetic acid, which accounted for 16.2, 24.8, and 15.7% of the chemical oxygen demand (COD) in BOAP, ROBOAP, and CFP, respectively. Propionic acid also represented a significant fraction of the feedstocks, at percentages of 3.2, 3.7, and 1.0% of the COD in BOAP, ROBOAP, and CFP, respectively. Phenol was also detected with concentration varying between 0.035 and 0.33% of the COD. However, there were also some dissimilarities concerning the substrates. CFP contained vanillic acid and lactic acid which represented 0.013% and 1.6% of the COD, respectively, and did not contain any furfural. HPLC was unable to confirm if lactic acid or vanillic acid were present in BOAP or ROBOAP. Some of these differences can be explained by the pretreatment. The stage fraction the ROBOAP was extracted from was designed to remove water and light oxygenated compounds [25], contributing to the high concentration of acetic acid found in the substrate. In contrast, BOAP was extracted from a pyrolysis process that was not fractionated into multiple streams [24]. CFP, being a fermentation product after acid and enzyme hydrolysis of corn stover, contained significant fractions of sugars and organic acids, with much smaller quantities of furans and phenolics [26, 27]. Other process conditions can affect the composition, as earlier studies have generated corn stover hydrolysis products or fermentation effluents with greater concentrations of furans and phenolics [28–30].

**Table 1:** Substrate COD before addition to reactors and compound concentration as a percent of the COD of the substrates

	BOAP	ROBOAP	CFP	PHE/ACE	ACE
Undiluted COD (g/L)	136.7	559.7	73.6	100	100
Acetic acid	16.2%	24.8%	15.7%	50%	100%
Furfural	0.91%	2.0%	ND	ND	ND
5-Hydroxymethylfurfural	0.31%	0.2%	0.10%	ND	ND
Vanillic acid	ND	ND	0.013%	ND	ND
Catechol	0.85%	0.2%	0.009%	ND	ND
Phenol	0.33%	0.3%	0.035%	50%	ND
Propionic acid	3.2%	3.7%	1.0%	ND	ND
4HB	ND	ND	ND	ND	ND
Lactic acid	ND	ND	1.6%	ND	ND
Total	21.8%	31.2%	18.5%	100%	100%

4-hydrozybenzaldehyde (4HB) was not detected in any of the substrates. The concentrations of acetic acid and phenol for the phenol/acetate blend (PHE/ACE) and acetate (ACE) were determined by weight measurement during their preparation, while the complex substrates’ composition was determined by HPLC

### Electrochemical performance of MECs under closed circuit conditions

**Fig. 1:**
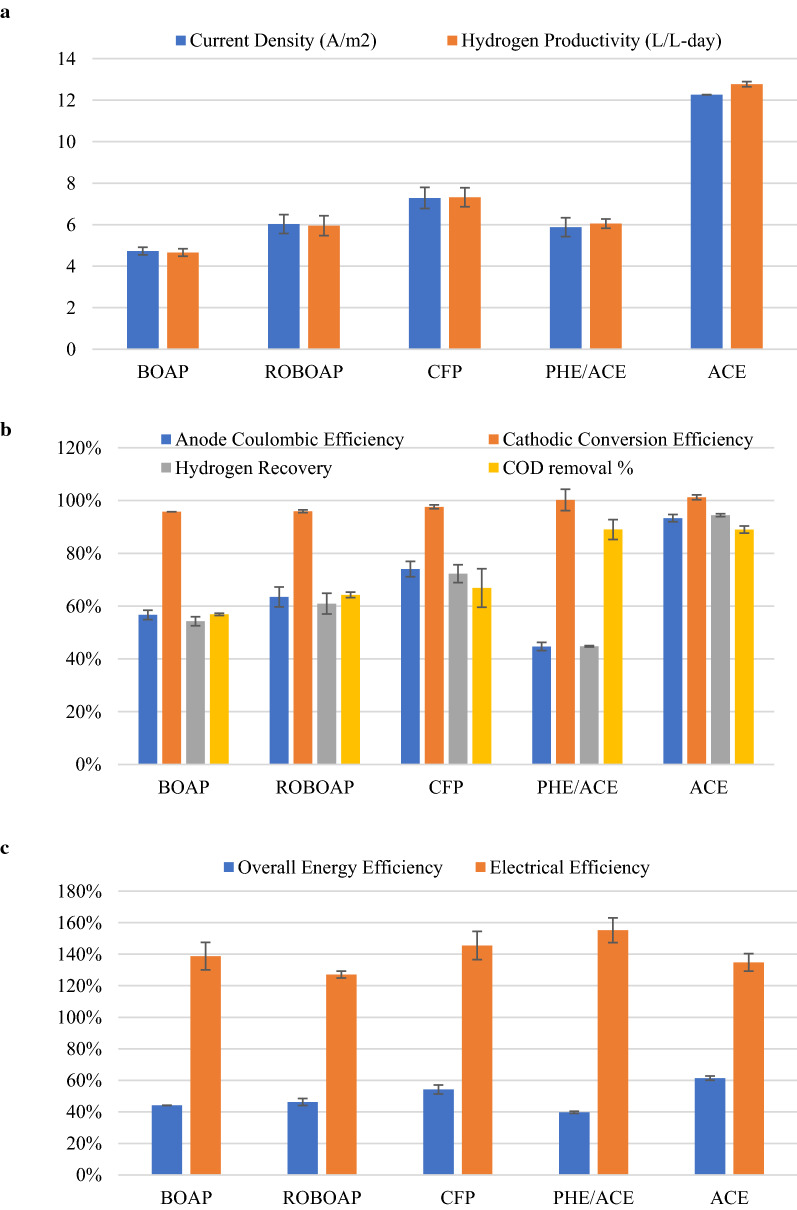
Electrochemical performance of MECs fed 10 g/L-day of each substrate. Substrates include switchgrass BOAP (BOAP), red oak BOAP (ROBOAP), corn stover fermentation product (CFP), equal fractions by COD of phenol and acetate (PHE/ACE), and acetate (ACE)

Among the three complex substrates investigated, CFP-fed MECs resulted in the highest performance. Figure 1 shows the electrochemical performance associated with the substrates tested. CFP-fed MECs produced an average current density of 7.3 ± 0.51 A/m^2^, while the lowest performance came from BOAP-fed MECs, generating 4.7 ± 0.18 A/m^2^. Correspondingly, CFP-fed MECs also had the highest hydrogen productivity of 7.3 ± 0.45 L/L-day, while BOAP-fed MECs produced the lowest hydrogen productivity of 4.7 ± 0.18 L/L-day. These findings agree with prior studies. Lewis et al. reported a current density of 4.5 ± 0.22 A/m^2^ and a hydrogen productivity of 4.3 ± 0.05 L/L-day with BOAP [24], while Satinover et al. reported current densities of 6.8 ± 0.33 A/m^2^ and a hydrogen productivity of 7.0 ± 0.50 L/L-day with CFP [22]. The CFP-fed MECs most likely out-performed the other complex substrates because of the concentration of easily degradable compounds and the low concentration of phenolics (see Table 1). Additional compounds not detected here also may have contributed to the performance. Satinover et al. showed high removal percentages of glycerol, arabinose, galactose, and xylose found in CFP occurred in MECs fed with CFP, which likely contributed to current production here [22]. BOAP, by contrast, had a smaller percentage of its COD represented as acetate, also containing larger concentrations of phenolics than CFP. ROBOAP-fed MECs, having a large fraction of the delivered COD coming from acetate, may have demonstrated lower performance due to the presence of other recalcitrant phenolics and furans. Other MECs using biomass feedstocks have not performed this well, several of which are orders of magnitude lower than what is produced here [5, 31, 32]. Acetate-fed MECs achieved the highest current density among the experiments, reaching 12.3 ± 0.007 A/m^2^ and a hydrogen productivity of 12.8 ± 0.12 L/L-day. Current density and hydrogen productivity were observed to be linearly correlated with the organic loading rate. Prior studies have shown similar correlations between organic loading, current density, and hydrogen productivity up to an organic loading rate of 10 g/L-day using biomass pyrolysis aqueous products [24, 33, 34], and up to 30 g/L-day using corn stover fermentation product [22].

Anode Coulombic efficiency varied more considerably from substrate to substrate, which was lowest when phe/ace was fed to MECs, reaching 44.7 ± 1.56%. Coulombic efficiency was highest for acetate-fed MECs, reaching 94.5 ± 1.37%. Of the complex feedstocks, CFP-fed MECs produced the highest Coulombic efficiency, while BOAP-fed MECs produced the lowest (Fig. 1b). This likely contributed to the hydrogen productivities observed. The substrate composition certainly contributed to this difference in Coulombic efficiency. CFP, being a more easily converted complex substrate due to its composition, was more directly converted to electrons than the more recalcitrant BOAP and ROBOAP. By contrast, the cathode conversion efficiency was high, exceeding 95% for the closed circuit experiments conducted. Because cathode conversion efficiency was consistently above 95%, cathode catalysis was not the largest limiting factor in MEC efficiency, but rather the anode’s ability to efficiently degrade organics.

Incomplete proton transfer observed by decreasing anode pH and high cathode pH was partially alleviated by recycling accumulated catholyte into the anode. However, additional pH adjustment was not required for acetate-fed MECs, NaOH addition was required for all other substrates in addition to catholyte recycling. Addition ranged from 0.2 ml every 24 h to 0.5 ml of 5 M NaOH, equivalent to adding approximately 14 mM/day of NaOH to maintain neutral pH. Substrate conversion, therefore, contributed to proton accumulation even after correcting for the incomplete proton transfer observed. Using an example, anaerobic acetogenic fermentation from glucose follows the overall reaction:

$$C_{6} H_{12} O_{6} \to 3CH_{3} COOH$$

Similarly, dark fermenters that convert glucose to acetate perform the following reaction:

$$C_{6} H_{12} O_{6} + H_{2} O \to 2CH_{3} COOH + 2CO_{2} + 4H_{2}$$

The acetate produced by dark fermentation is then metabolized via the exoelectrogens and MEC cathode to drive the reaction:

$$CH_{3} COOH + 2H_{2} O \to 2CO_{2} + 4H_{2}$$

CO_2_ reacts with water to form carbonic acid, which continues to lower the pH of the anode liquid medium. The drop in pH caused by carbonic acid accumulation can be overcome partially by degassing the anode liquid media, which was not performed during the experiments, except at the beginning when the anode liquid medium was sparged. Continuous degassing would have prohibited the anode gas analysis needed for the electron balance. However, future MEC designs may benefit from degassing the anode liquid to assist in neutralizing the anode pH. In addition, while glucose can convert to CO_2_ and H_2_O in MECs, other metabolic processes may be occurring in MECs that do not result in complete compound oxidation. For these metabolic processes, acids and protons will accumulate even if perfect proton transfer to the cathode and subsequent conversion to hydrogen occurs. Buffer use and pH control in MECs will be a necessity for complex feedstocks containing substrates that acidify upon degradation. For the complex feedstocks used here, pH adjustment would still be required for MECs even with ideal mass transfer.

**Fig. 2:**
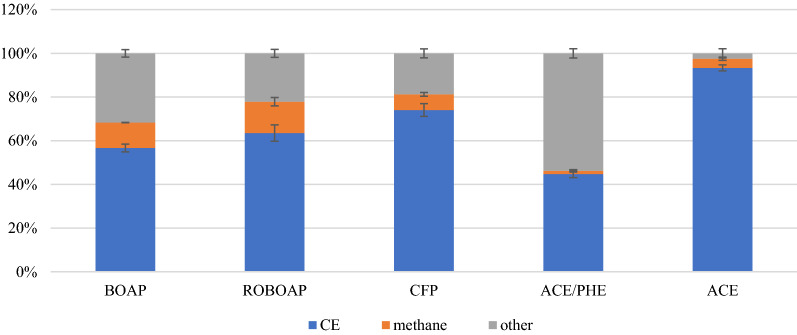
Electron balance results showing the percentage of COD that corresponded to electrons that contributed to current (CE), methane, or other sinks. Substrates include switchgrass BOAP (BOAP), red oak BOAP (ROBOAP), corn stover fermentation product (CFP), equal fractions by COD of phenol, and acetate (PHE/ACE), and acetate (ACE)

The electron balance determined that methane accumulation occurred for each of the substrates tested, but to a varying degree. These results are shown in Fig. 2. Methane was detected in the headspace of the anode ranging from 1.5 to 14.4%, and was largest in MECs fed with ROBOAP, representing 14.4 ± 1.93% of the electrons extracted from COD. In acetate-fed reactors, diversion of electrons to methane was small (4.2 ± 0.75%). Acetate-fed reactors also had very little of the substrate diverted to other sinks (2.5 ± 2.12% towards other electron sinks). This suggests that acetate was minimally converted to methane via acetoclastic methanogenesis. The phe/ace fed MECs showed the largest diversion of electrons to unknown sinks, representing 53.8 ± 2.13% of the total converted COD. Undefined sinks have been attributed to extracellular storage by Freguia et al. [35]. It is possible that phe/ace fed MECs diverted more of the substrate to storage. However, HPLC showed that another mechanism was taking place: substrate adsorption. This mechanism is further discussed in the following section.

Electrical efficiency ranged between 127.1 and 155.2%, with ROBOAP-fed MECs producing the lowest electrical efficiency, and phe/ace-fed MECs producing the highest. Thus, more energy was recovered as hydrogen than what was delivered as electrical current. By contrast, overall efficiency was highest for acetate-fed MECs at 61.4 ± 1.34%, and lowest for phe/ace-fed MECs at 39.8 ± 0.67%. This difference in overall energy efficiency is largely determined by the COD removal percentage and the anode Coulombic efficiency. Because the Coulombic efficiency of the phe/ace experiments was also lowest despite high COD removal percentages (89.0 ± 3.76%), this translated to a smaller overall energy efficiency. Acetate-fed MECs not only degraded high percentages of delivered COD (89.9 ± 3.77%) but also had higher Coulombic efficiencies as shown earlier. Combined with the electron balance results shown in Fig. 2, it is apparent that the substrate conversion efficiency largely dictated the overall energy efficiency of the MECs.

### Substrate conversion under closed circuit conditions

**Fig. 3:**
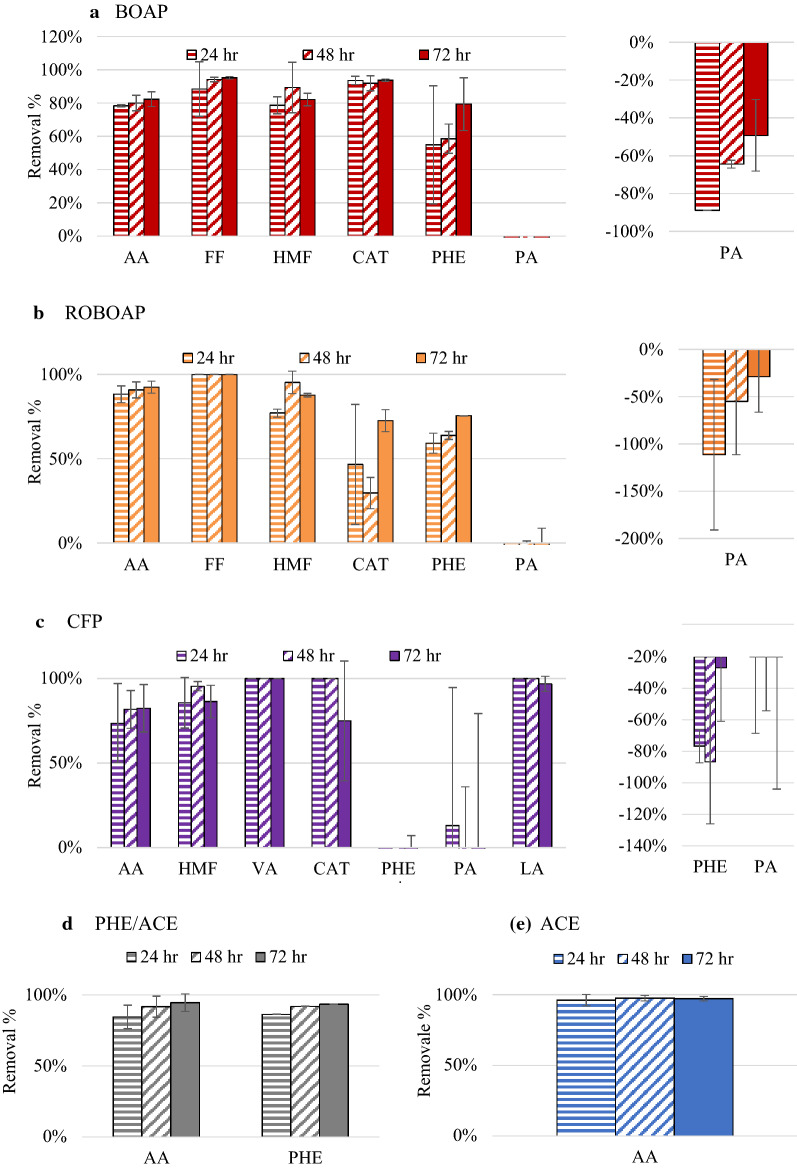
Compound removal percentage of MECs fed 10 g/L-day of each substrate. Substrates include switchgrass BOAP (BOAP), red oak BOAP (ROBOAP), corn stover fermentation product (CFP), equal fractions by COD of phenol and acetate (PHE/ACE), and acetate (ACE). Compounds include acetic acid (AA), furfural (FF), 5-hydroxymethylfurfural (HMF), vanillic acid (VA), catechol (CAT), phenol (PHE), propionic acid (PA), and lactic acid (LA)

Nine different compounds were quantified in the reactors via HPLC to understand the conversion of the substrates, where high removal percentages of some compounds were observed. Figure 3 shows these trends for each of the compounds tested. Acetate was removed above 75% for all substrates tested. Propionic acid accumulated for the substrates tested, with the lowest percentage of removal being − 111.3 ± 79.6% for ROBOAP-fed MECs after the first 24 h. Its removal rate increased to − 28.8 ± 37.6% as the experiment progressed. A similar trend was observed with BOAP-fed MECs, while in CFP-fed MECs the accumulation decreased only after 72 h. Propionic acid is a potential intermediate in the metabolism of complex substrates, and is a common fermentation product [36]. The initial accumulation of propionic acid indicates that there was a delay in the starting metabolism of some of the microbes in these MECs. Furthermore, the communities were not as capable of degrading propionic acid as reported previously, where Lewis et al. reported propionic acid degradation percentages above 60% using the same switchgrass BOAP feedstock [24]. How it accumulates or degrades in MECs operated on complex feedstocks is not well understood. These results show that propionic acid accumulation is largely contingent on the substrate type and the community. The exact pathways used by the microbial community or the precursors from which it is derived have not been determined.

During closed circuit experiments with acetate-fed MECs, phenol appeared in the anode liquid medium as a function of time, resulting in concentrations of 5.29, 7.64, and 9.98 mg/L at the 24, 48, and 72 h, respectively. Scaled to the anode volume, this represents an approximate maximum desorption rate of 43.8 mg/L-day. Because no phenol was added to the MEC during this experiment, this finding suggests that phenol was retained in the anode likely via sorption to the felt. Much of this sorbed phenol likely came from the 2.3 g/L phenol batch addition experiment conducted before the experiments with acetate. Even with a recovery period afterwards that was more than 3 weeks long and three anode liquid medium replacements, phenol was still apparent in the anode liquid medium. Phenolic compound adsorption to porous carbon materials, such as activated carbon, is well documented [37], and is not unheard of in other bioelectrochemical systems (BESs). Hejazi et al. capitalized on phenol adsorption for use in a granular activated carbon anode in a microbial fuel cell (MFC), and achieved a removal efficiency of 95% after 72 h and reached a Coulombic efficiency of 45.77% [38]. Zhang et al. also suggested that phenol adsorption occurred when fed to their BES, which achieved more than 95% removal of phenol and Coulombic efficiencies as high as 27.3% [39]. While the MECs used here did not use activated carbon, we observed a similar removal percentage of phenol with the phe/ace fed MECs, reaching a removal percentage of 93.5 ± 0.08% in 72 h. Other studies that have experimented with phenol-fed BESs have found Coulombic efficiencies lower than 10% [40, 41]. In this study, the Coulombic efficiency for phe/ace fed MECs was below 45% despite HPLC suggesting that more than 90% of the phenol and acetate was removed after the experiment. Assuming acetic acid was the only compound that contributed to current in the phe/ace blend fed to the MECs, the acetic acid would have generated a Coulombic efficiency of 84.2 ± 1.00%. The MECs fed with acetate had higher Coulombic efficiencies than this, reaching 93.3 ± 1.37%. Thus, a significant fraction of phenol that was removed may not have contributed to current or was not degraded biologically. This is futhur supported by the results in the Electrochemical performance of MECs under open circuit conditions section.

It is also possible that other compounds that are removed at large percentages may be sorbing in some capacity. Large removal percentages for some compounds, including vanillic acid, 5-hydroxymethylfurfural, and furfural, were observed for all substrates tested, exceeding 75%. Sorption is electrode dependent. Furfural, 5-hydroxymethylfurfural, and vanillic acid, did not adsorb to the carbon felt used in MECs according to Zeng et al. [16]. By contrast, furans and phenolics have been shown to adsorb to activated charcoal that follow Langmuir and Freundlich isotherm models (*R*^2^ > 80%) [42]. Sorption without conversion would partially explain the lower Coulombic efficiencies in the complex feedstocks compared to acetate fed MECs. It is possible that the compounds detected besides phenol contributed to current only minimally regardless of the mechanism of removal. Zeng et al. [43] performed an electron balance on MECs using phenolics including syringic acid, vanillic acid, 4-hydroxybenzoic acid, and found that only vanillic acid and 4-hydroxybenzoic acid had electron equivalents that did not contribute primarily to current. The compounds analyzed by Zeng et al. [43] would probably not be the only sorbing compounds, as the total COD contributed by the phenolic compounds detected in the complex substrates represented less than 6% of the substrate COD according to HPLC. Even if they did not convert effectively, these compounds would not have a significant impact on performance. Other compounds present in these complex feedstocks were not tested but will need to be examined in future studies, as the implications for the functionality of the electrode material on MEC performance are significant. Fermentation has been deemed limiting in MECs fed with BOAP [13], and this apparent limitation may be contributed by the immobilization of compounds sorbed to the anode.

### 16S rRNA gene sequencing results

**Fig. 4:**
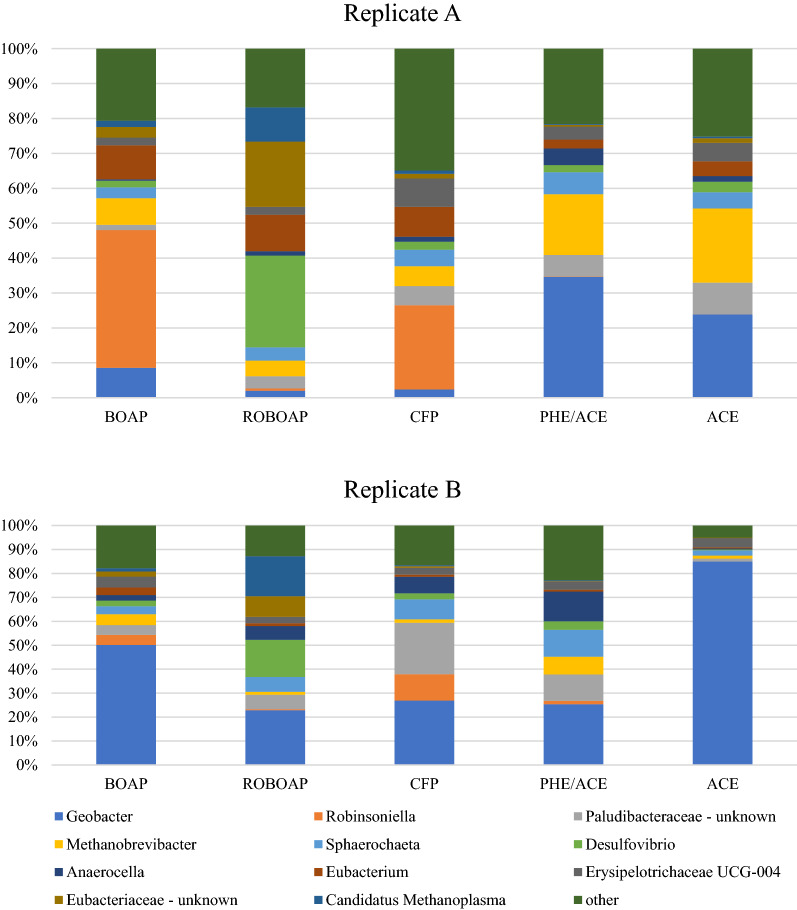
16S rRNA sequencing of reactor replicates **a** and **b**. Reactor **a** and **b** had very different microbial compositions despite coming from the same inoculum source and being grown under identical conditions. Substrates include swtichgrass BOAP (BOAP), red oak BOAP (ROBOAP), corn stover fermentation product (CFP), equal fractions by COD of phenol and acetate (PHE/ACE), and acetate (ACE)

The most dominant genus on average across all conditions tested was *Geobacter*, however the relative abundance of *Geobacter* was inconsistent when comparing replicates. Figure 4 shows the bar charts for the 16S rRNA gene sequencing results. As shown by the figure, replicate B had significantly more abundance of *Geobacter* than replicate A for the substrates tested. When MECs were fed with acetate, replicate B had 84.97% of the population represented by *Geobacter*. *Geobacter* is a commonly known exoelectrogenic genus, and while dominant in replicate B, it was under-represented in replicate A during growth on complex feedstocks. The average relative abundance of *Geobacter* in replicate A across all substrates tested was 14.3%, while it was 42.1% in replicate B. When fed with phenol and acetate, both replicates had significant *Geobacter* populations, reaching 34.6% of the OTUs in A and 25.4% of the OTUs in B. Based on these results, the current density was not found to correlate strongly with relative abundance of *Geobacter* (*R*^2^ = 0.20). The results suggest that *Geobacter* was not entirely necessary for achieving high performance in MECs using complex substrates.

In replicate A, other genera may have played an exoelectronic role. The abundance of *Palundibacter* correlated strongly with current density via Pearson correlation (*R* = 0.89). However, the correlation associated with replicate A was not consistent with replicate B. The *R* value in replicate B between current density and an unknown genus of Paludibacteraceae was − 0.31. This discrepancy was found to be true for the other prominent genera identified. Furthermore, correlations across both data sets were found to be weak, and did not exceed 0.5 for any genus tested (see Additional file 1: Table S1). Correlations were also found to be sensitive, changing significantly when samples were removed from the analysis. Thus, individual OTUs were not found to be a strong deterministic factor for electrochemical performance, indicating functional redundancy. Similar conclusions were reported by Ruiz et al. [9] and Miceli et al. [10], which are now expanded to complex feedstocks starting with a single inoculum source.

The work here also suggests that diversity is not a good predictor of performance either, as performance for either reactor did not correlate with calculated diversity metrics (The diversity metrics are shown in Additional file 1: Table S2). Low correlation between diversity and performance has been reported by Stratford et al., who showed that *R* values were consistently below 0.65 when correlating diversity and power density in MFCs fed with glucose [44]. The correlations in this study were even weaker. R values across both replicates were − 0.28, − 0.53, and − 0.48, for Chao1, Simpson, and Shannon indices, respectively. Data from acetate-fed MECs was seen to skew these results due to the high current density and diversity variability between the replicates. When acetate-fed MEC data was omitted, Pearson correlation coefficients changed to 0.05, 0.71, and 0.84 for Chao1, Simpson, and Shannon indices, respectively. Acetate results should not be ignored, as device performance was similar across the replicates despite their differences in community structure. Therefore, these results suggest that alpha diversity is only marginally (if at all) predictive of current density in MECs fed with complex feedstocks, and, therefore, performance.

Even though the correlations between community structure and performance were not very strong, the 16S rRNA gene sequencing can still be useful in explaining the observations. *Robinsoniella* was present in significant quantities for both replicates and was represented largely in MECs fed with BOAP and CFP. The sequencing results allowed for species identification of this genus, indicating that the *Robinsoniella* was actually *R. peoriensis. R. peoriensis* has been shown to grow on several poly and monosaccharides, including, amygdalin, arabinose, cellobiose, fructose, glucose, maltose, lactose, raffinose, starch, trehalose, xylan and xylose, according to Cotta et al. [45]. As mentioned earlier in the Results and discussion section, CFP has been shown previously to contain significant fractions of xylose and arabinose, and was also found in MFCs fed with the same substrate previously [22, 26, 27]. Lachnospiraceae, the family that *R. peoriensis* belongs to, was also found in MECs fed with BOAP [24]. Thus, these results are consistent with prior reports. Interestingly, the abundance of *R. peoriensis* declined when MECs were fed with ROBOAP, phe/ace, and acetate, to less than 2% of the relative OTUs. For acetate and/or phenol–fed MECs, this is expected, as there is little or no evidence to suggest that *R. peoriensis* can use either phenol or acetate. While the substrates were not analyzed for sugar content during this work, previous analyses suggested that ROBOAP contained smaller amount of sugars compared to BOAP and CFP [25, 26, 46], that is needed for *R. peoriensis* enrichment.

While *Geobacter* was the dominant exoelectrogenic genus observed, it was not the only exoelectrogen detected. *Desulfovibrio* were also determined in significant quantities in this study, particularly in the MECs fed with ROBOAP, exceeding 15% of the relative abundance among the replicates. Traditionally, *Desulfovibrio* is a sulfate reducing bacterial genus, however a species of this genus has been shown be exoelectrogenic without the need for sulfate [47]. Furthermore, the fraction of the pyrolysis oil that represented ROBOAP contained low sulfur concentration, at approximately ~ 0.03 g/g- of the ROBOAP [25]. The reason for enrichment of this microbe likely stems from the type of carbon in the ROBOAP sample. Identifying that exact source within the complex substrate is difficult and out of scope of this study.

Another abundant microbe was an unknown genus of Paludibacteraceae. The only identified genus of Paludibacteraceae is *Paludibacter*, which contains species that are fermenters that produces propionic and acetic acid [48, 49]. *Paludibacter* were also found in long tubular MFCs fed with sucrose [11]. This may explain the propionic acid accumulation observed from the complex substrates in open circuit conditions. However, the final products of Paludibacteraceae are not the same as other fermenters found in these MECs. Dark fermentation may also be taking place. Potential dark fermenters were also found in MECs. A strain of *Sphaerochaeta* has been found to contain species that produce hydrogen from glucose fermentation, while it could also grow on other sugars including maltose, ribose, and xylose [50]. Sun et al. also determined that *Sphaerochaeta* was enriched (6% relative abundance) in MFCs fed with a phenol-rich complex feedstock, pyroligneous liquor, which removed 84% of the phenol delivered [51]. Together, this suggests that the *Sphaerochaeta* found in the MECs used in this study may have produced hydrogen during phenolic degradation while consuming other sugars not detected here. Another potential dark fermenter is *Eubacterium*, which was also found in the highest abundances during ROBOAP feeding. Wallace et al. suggested that a species of *Eubacterium*, *E. pyrovativorans*, produced hydrogen from pyruvate and lactate, although growth on lactate was limited [52]. It has also been shown to use amino acids, as it is not saccharolytic [53]. For any of the dark fermenters detected, the MECs could enrich a co-culture of hydrogenotrophic organisms.

Methanogens were also present in the anode samples, represented primarily by non-acetoclastic methanogens, which further supports the results of the electron balance in Fig. 2. *Methanobrevibacter* sp. was found to be the most abundant methanogen across all substrates tested, and whose population varied considerably depending on the replicate observed. In replicate A, *Methanobrevibacter* were most prominent in MECs fed with acetate (21.2% relative abundance) as well as when fed phe/ace, while in Replicate B, *Methanobrevibacter* represented 7.4% with phe/ace as the substrate, but less than 2% with acetate as the substrate. *Methanobrevibacter* has species that produce methane from hydrogen, such as *M. smithii*, which is often studied due to its presence in humans [54]. The source of this hydrogen could come from several places but is unlikely to be due to back diffusion from the cathode. The cathode conversion efficiency exceeded 95% across substrates, so it is unlikely that the hydrogen used by methanogens came from cathode. According to the electron balance (Fig. 2), methane represented a much larger electron sink than what could be attributed to losses in cathode conversion efficiency (Fig. 1b). Therefore, dark fermentation likely contributed to its enrichment. *Methanobrevibacter*’s presence is also unusual for acetate fed MECs given how little methane was created from COD removal. For acetate-fed MECs, such high abundances seen in replicate A would not be expected. It is possible that compounds such as phenol, which partially sorbed to the felt, continued to enrich this microbe even without additional carbon sources being provided.

*Candidatus Methanoplasma* was also found but mainly in MECs fed with ROBOAP. Unlike *Methanobrevibacter*, it is unlikely that *Candidatus Methanoplasma* can make methane from CO_2_ and H_2_ alone. This genus belongs to a group of microbes known as seventh order methanogens, which have been reported to be hydrogen-dependent methylotrophs [55]. Methylotrophs consume compounds such as methanol, which was not investigated for any of the substrates or MEC effluents, however other methylated compounds have been reported in the stage fraction representing ROBOAP [25]. Thus, if methanol was the substrate that promoted *Candidatus Methanoplasma* enrichment, it was likely an intermediate from degradation of higher order methylated compounds. This may also explain why methane represented a larger diversion of elections in ROBOAP-fed MECs than with the other substrates. As *Candidatus Methanoplasma* populations increased in both reactor replicates from the starting communities in BOAP-fed MECs, it produced more methane.

The results from the 16S rRNA gene sequencing demonstrate that the substrate has a definite impact on community composition, but it is not co-related with performance. The differences observed amongst replicates which were fed the same carbon source could be attributed to subtle differences in the MECs that are not well understood, as the initial growth procedures were identical amongst both replicates and the inoculum source was also the same. It appears that communities found in MECs exhibit functional redundancy. There are many species in MECs that are known exoelectrogens, and there are an even greater number of fermenters that could be present in the community that fill similar roles. Further investigation on the established function of the genera discovered will help support this hypothesis.

### Electrochemical performance of MECs under open circuit conditions

**Fig. 5:**
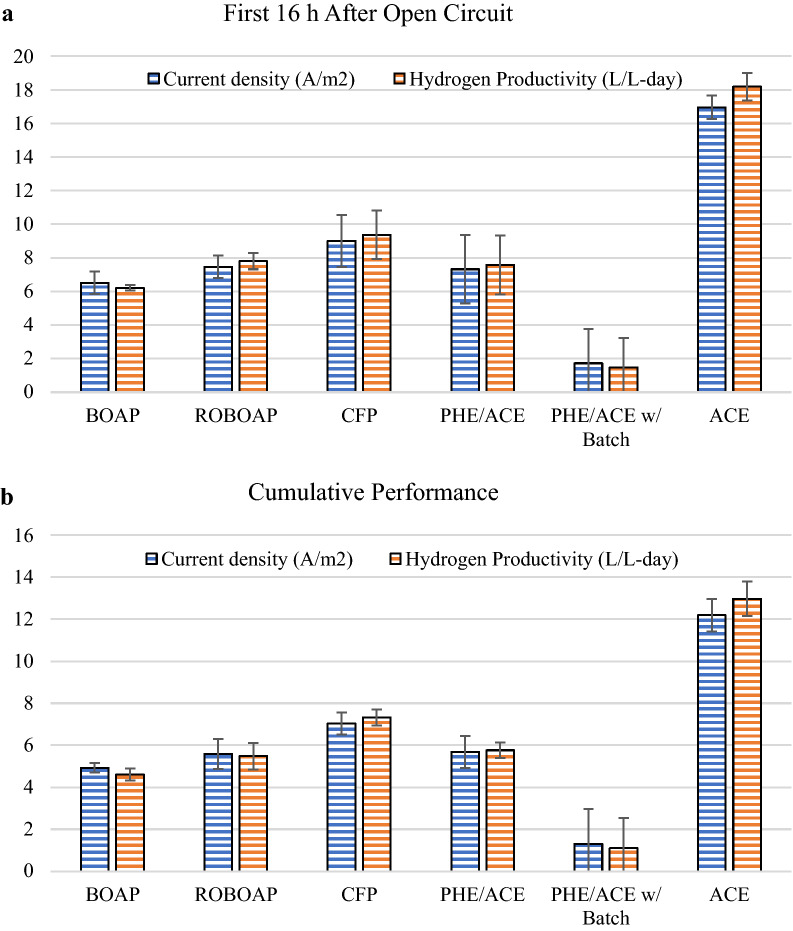
Electrochemical performance of MECs fed 10 g/L-day of each substrate 16 h after open circuit conditions (**a**), and the cumulative performance including the open circuit data (**b**). Substrates include BOAP, red oak BOAP (ROBOAP), corn stover fermentation product (CFP), equal fractions by COD of phenol and acetate (PHE/ACE), equal fractions by COD of phenol and acetate fed 2.3 g/L batch phenol (PHE/ACE w/Batch), and acetate (ACE)

When the MECs were operated under open circuit conditions, only negligible amounts of hydrogen were produced, as expected. The current density and hydrogen productivity was assessed after reinstatement of closed circuit conditions and was found to be higher in the following 16 h. However, when averaged over the whole duration of the experiment, it was similar to the uninterrupted closed circuit experiments. These trends are shown in Fig. 5a, b, respectively. Acetate-fed MECs had the largest productivity, having an average current density of 12.2 ± 0.78 A/m^2^ and an average hydrogen productivity of 13.0 ± 0.83 L/L-day, which is similar to the performances found in uninterrupted closed circuit conditions. The worst performance occurred with BOAP-fed MECs, which had an average current density and hydrogen productivity of 4.9 ± 0.22 A/m^2^ and 4.6 ± 0.29 L/L-day. While the current density was slightly larger for BOAP-fed MECs in the prior experiments, the error associated with the measurements suggests that the difference in the average current density and hydrogen productivity between both sets of experiments were not statistically different (*p* > 0.05). This was found to be true for all the substrates tested. The largest current density and hydrogen productivity during the 16 h after closed circuit conditions was demonstrated by acetate-fed MECs at 17.0 ± 0.70 A/m^2^ and 18.2 ± 0.81 L/L-day, respectively. The lowest was demonstrated by BOAP-fed MECs, at 6.5 ± 0.67 A/m^2^ and 6.2 ± 0.15 L/L-day for current density and hydrogen productivity, respectively. This suggests that fermentation continued in the MECs without exoelectrogenesis of the intermediates generated under the open circuit conditions. Once closed circuit conditions were applied, exoelectrogenesis resumed, removing the accumulated compounds and generating current. This result also suggests that the MECs have higher capability to generate current as well as hydrogen if suitable substrates are available. For some substrates, the opposite observation was made. Inhibition was demonstrated in fed-batch conditions using 2.3 g/L of added phenol to phe/ace-fed MECs. These MECs performed the worst of all the conditions tested after closed circuit conditions were applied, reaching average current densities and hydrogen productivities of 1.3 ± 1.66 A/m^2^ and 1.1 ± 1.44 L/L-day, respectively. This loss in performance was more than the 50% decrease in performance originally discussed by Zeng et al. [17] at the same batch addition of phenol used here. Inhibition can, therefore, play a role in even robust high performing communities.

### Substrate conversion under open/closed circuit conditions

**Fig. 6:**
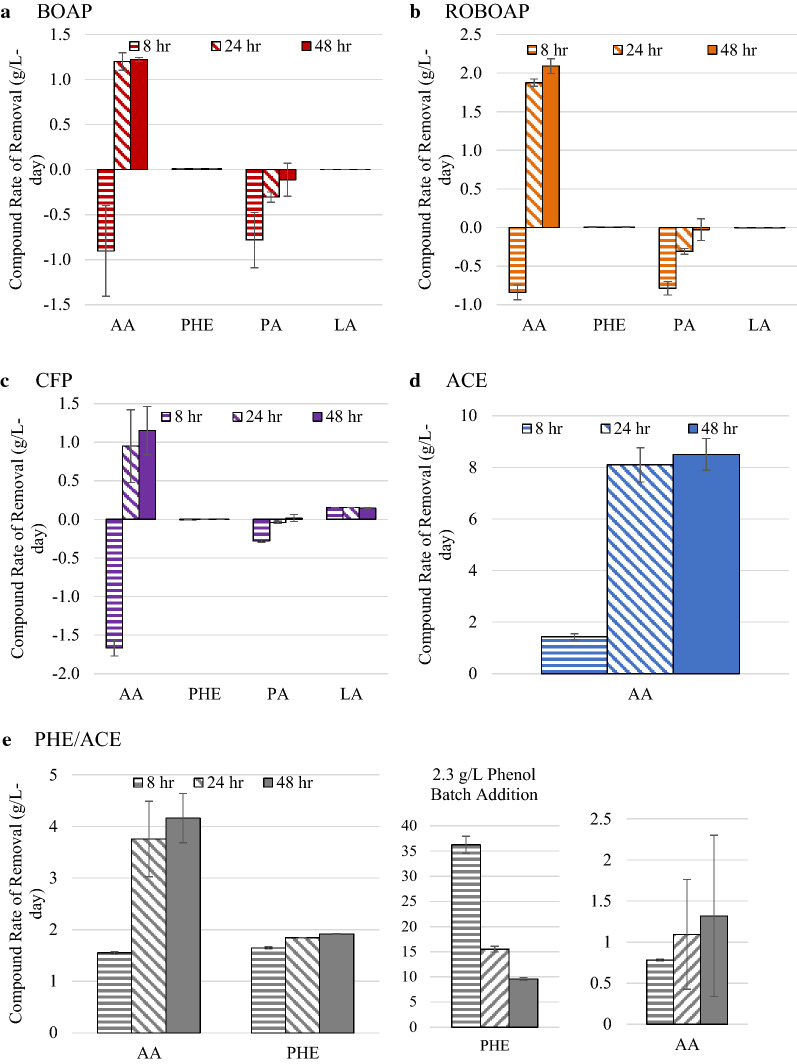
Compound removal rates during open circuit conditions (8 h) and during closed circuit conditions at 24 h and 48 h time points. Compounds shown include acetic acid (AA), phenol (PHE), propionic acid (PA), and lactic acid (LA). Substrates include switchgrass BOAP (BOAP), red oak BOAP (ROBOAP), corn stover fermentation product (CFP), acetate (ACE), and equal fractions by COD of phenol and acetate (PHE/ACE). Fig. 6e also includes the conditions where 2.3 g/L of phenol were added, labeled as  2.3 g/L Phenol Batch Addition

Acetate is frequently used as model substrate in MECs for exoelectrogenesis, and therefore, its conversion in the MEC is very important. Each of the experiments using complex feedstocks resulted in some accumulation of acetic acid. Fig. 6 shows the results from this set of experiments. The largest accumulation rate of acetic acid occurred when MECs were fed with CFP, which was at a rate of 1.7 ± 0.10 g/L-day, while ROBOAP-fed MECs accumulated the least amount of acetate at 0.8 ± 0.10 g/L-day. When MECs were switched to closed circuit conditions, acetic acid removal percentages returned to values that were similar to closed circuit conditions after 24 and 48 h. Net acetic acid removal, taking the difference between the acetic acid removal rates in closed circuit and open circuit conditions, was largest for ROBOAP at 2.93 ± 0.0004 g/L-day. Acetate was, therefore, rapidly consumed. This supports the idea that fermentation is limiting for complex feedstocks [13], generalizing this finding to other types of complex feedstocks. Net acetic acid removal correlated slightly with current density and hydrogen productivity. The correlation between average net acetic acid removal and current density was found to have an *R*^2^ value of 0.79. The whole data set appeared skewed from the acetate-fed MEC data, as the *R*^2^ decreased when the data for the acetate-fed MECs was excluded, dropping to a value of 0.61. If acetate were the only compound that contributed directly to exoelectrogenesis, we would expect this correlation to be stronger. Other compounds, including organic acids, sugars, and alcohols, have been used as electron donors for growth using Fe(III) as the electron acceptor [56]. However, Fe(III) reduction is not identical to anode respiration, and such reduction has been shown to be result in different gene clusters being expressed for each electron acceptor [57]. Therefore, there are other compounds that may be contributing directly to exoelectrogenesis that may not occur in other experimental conditions, and this needs further exploration.

The other VFA tested was propionic acid, which accumulated more in open circuit conditions than the closed circuit conditions. The largest accumulation rate for propionic acid was with MECs fed with ROBOAP, which was a rate of 0.6 ± 0.14 g/L-day. Interestingly, CFP-fed MECs accumulated the least amount of propionic acid during open circuit conditions, reaching an accumulation rate of only 0.27 ± 0.012 g/L-day. This amount of accumulation was proportionally large and represented more than a 400% increase in propionic acid accumulation from what was delivered. Comparing the total accumulation rates of both organic acids, CFP accumulated the most organic acid at a combined rate of 1.95 g/L-day, while ROBOAP accumulated the least at 1.63 g/L-day. Accumulation percentages of some compounds not traditionally considered substrates for exoelectrogens, such as catechol and phenol, were also observed at larger accumulation percentages during open circuit conditions than closed circuit conditions when fed with CFP. However, as discussed earlier in this study, the quantities of these compounds delivered were small enough that they likely did not contribute to current as prominently as acetic acid or other compounds not determined here. Here, it is unlikely that exoelectrogens were the dominant microbes that consumed lactic acid, as CFP-fed reactors (the only substrate, where lactic acid was detected) did not accumulate lactic acid when operated in open circuit conditions. Fermenters represented a much larger fraction of the community, with correspondingly higher metabolic diversity as supported by the 16S rRNA sequencing data shown earlier.

Acetic acid was also removed initially when fed to reactors in open circuit conditions for both phe/ace and acetate-fed MECs. There are several potential explanations for this. One may be sorption to the anode. Lee and Park determined adsorbance isotherm curves for acetate using activated charcoal [42], so this mechanism might be possible at first glance. However, the Coulombic efficiency for the acetate fed MECs under closed circuit conditions exceeded 90%, and acetate was consumed rapidly in closed circuit conditions. This suggests that even if this mechanism were true, only a small fraction would be sorbing to the felt without being converted under the conditions tested. Thus, it is unlikely that this mechanism is the primary explanation for the acetic acid removal observed in open circuit conditions. A more likely explanation may come from the capacitive properties commonly found in BESs. BESs exhibit internal capacitance and behavior associated with other circuit elements, as demonstrated by electrochemical impedance spectroscopy [58–60]. Furthermore, exoelectrogenic microbes can also exhibit capacitance in the external conductive network created by microbial biofilms [61]. In order for exoelectrogens to create charge, a carbon source must be available. Exoelectrogenic biofilms and MECs may, therefore, exhibit capacitance during open circuit conditions, resulting in a loss of acetate. This is further supported by the amount of acetate removed during experiments, where MECs were fed phe/ace and acetate. In the 8 h of open circuit conditions, the removal rates were 1.56 ± 0.02 g/L-day and 1.43 ± 0.11 g/L-day for phe/ace and acetate-fed MECs, respectively. It is possible that the MECs reached their threshold for capacitance at this removal rate. Testing additional loading rates of acetate may further support this explanation.

### Principal component analysis loading plot results

**Fig. 7:**
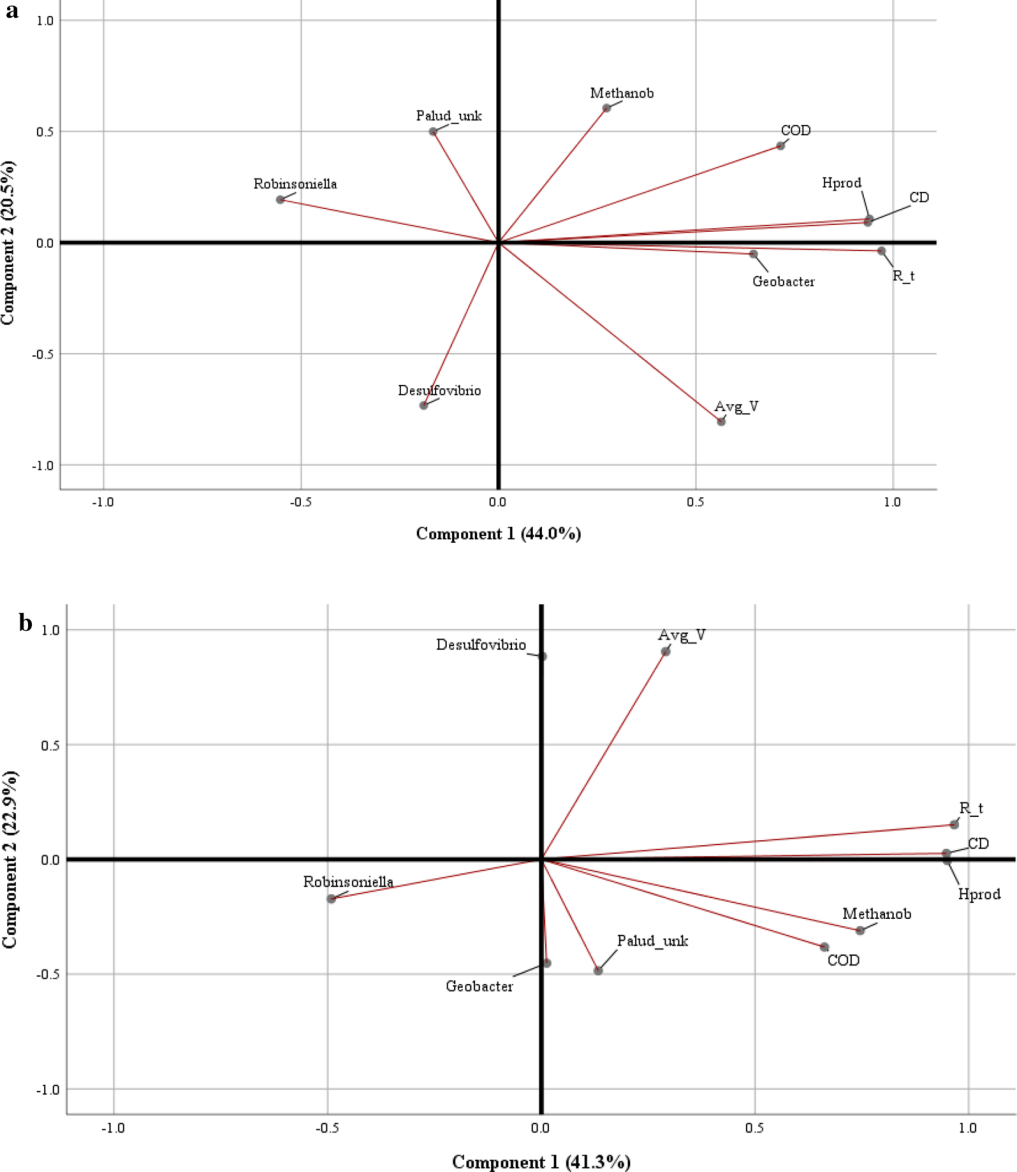
PCA loading plot with all data (**a**) and without replicate **b**-acetate-fed MECs (**b**). Variables used in the analysis include Current Density (CD), Hydrogen Productivity (Hprod), Net Acetic Acid Removal, *R*_*t*_ (*R*_*t*), AverageWhole Cell Voltage (Avg_*V*), COD Removal  % (COD), and relative abundances of the following microbe genera: Geobacter, Robinsoniella, Paludibacteraceae–unknown (Palud_unk), Methanobrevibacter (Methanob), and Desulfovibrio

Principal Component Analysis (PCA) was carried out using the five most dominant microbial genera across all the systems. These included the relative abundances of: *Geobacter*, *Robinsoniella*, *Paludibacteraceae*–*unknown*, *Methanobrevibacter*, and *Desulfovibrio*. Key performance metrics were also included. The loading plot from PCA is shown in Fig. 7a for all of the data collected. As shown, *Geobacter* was the strongest correlated genus with the electrochemical performance metrics (current density, hydrogen productivity), which were otherwise tightly grouped when all of the data was included. *Geobacter*’s correlation was most likely skewed due to the high abundance in replicate B when fed with acetate. When the same analysis was performed without the data from replicate B when fed with acetate (Fig. 7b), the results were much different. Microbes and performance do not seem to be strongly correlated. *Robinsoniella* seemed to be negatively correlated to performance. Whether or not this means that *Robinsoniella* would be present only on poorly converted substrates is unknown. However, it may be fair to assume that some fermenters such as *Robinsoniella* are not as active as others that may contribute to faster conversion of organics to current or MEC efficiency. Alternatively, there may be compounds present in complex substrates that are inherently recalcitrant that only *Robinsoniella* is capable of degrading. If MECs are fed with recalcitrant substrates, which may be the case, since BOAP contains many such compounds like phenolics and furans [13, 24], microbes that poorly contribute to current or substrate degradation may be enriched.

Performance metrics including current density, net acetic acid removal rate, and hydrogen productivity were grouped closely together. By contrast, COD removal % and average voltage were not, as they appear to be anti-correlated to each other. This is surprising considering the organic loading rate was equal for all of the substrates used. As more COD was removed, current densities should increase and average voltages should rise. This analysis shows that the relationships between community structure and substrate degradation are much more complex and require further investigation. Accumulation of various intermediates shown in this study indicate that fermentation and exoelectrogenesis are not always in balance and are dynamic in nature. This could lead to the unexpected co-relations observed via PCA. Modeling of fermentation, exoelectrogenesis, and the growth rate of the two class of organisms is required at a minimum to understand these dynamics.

Interestingly, *Methanobrevibacter* was associated with both components shown in both analyses, indicating that it had somewhat of a grouping with performance. Given the presence of dark fermenters, *Methanobrevibacter* may have assisted in accelerating higher order compound degradation, consuming accumulated hydrogen that might have otherwise caused product inhibition to the fermenters. By contrast, *Methanobrevibacter*’s presence suggests that the exoelectrogens could not metabolize the available substrate quickly enough to fully outcompete this methanogen’s use of hydrogen. *G. sulfurreducens* has been shown to oxidize hydrogen or acetate under iron reducing conditions, however dissolved hydrogen has been shown to slightly inhibit acetate oxidation [62]. *Desulfovibrio* also seemed more strongly related to the second component than the first in both loading plots, which suggests that it is not correlated with MEC performance. This confirms that its presence was not necessary to achieve high performance but must be serving a different function, specifically related to conversion of ROBOAP.

The total variance represented by the PCA plots accounts for 64.5% of the dataset’s variance with all of the data (Fig. 7a), and 64.2% of the variance when the acetate-fed replicate B data was excluded (Fig. 7b). As shown, when individual samples were pulled from the analysis, the loading plots changed dramatically. This analysis, therefore, does not define the relationships between variables as strongly as it would if the components represented more of the variance. This further supports the idea that community structure cannot be used to accurately predict performance. While the performance did not correlate with community structure, the study clearly demonstrated the impact of feedstock on the microbial community grown from the same inoculum.

### Study limitations and future work

The work here suggests that community structure is not necessarily correlated with compound removal or performance. However additional study is necessary to fully confirm the results shown. Effectively expanding these results will mostly rely on expanding the methods, which requires additional resources and time that were not available during this study. For instance, this study only used two replicates. This concession was made primarily due to instrument availability, personnel availability, and timing required to operate the MECs. Basic operating procedures, such as anode liquid medium changes, pH adjustments, samples collection, available potentiostat channels, and bench space contributed to significant time, effort, and resource requirements that limited the potential depth of the experiments. These limitations could be partially resolved through process controls, improved reactor design, and new operational procedures that will not only improve the depth of data collection but will also benefit the operation of future MECs.

Several other areas worth exploring include expanding the depth of the variables examined. This study targeted complex feedstocks, which contain numerous classes of compounds. Only three complex feedstocks and two pure compounds (acetate and phenol) were investigated, and experimental data could have benefitted from examining more substrates. Looking at alternative feedstocks readily available as waste could represent a new point of interest. In addition, adaptation time and procedures were constant, and changes to adaptation time may affect the resultant community structure. Electrochemical analysis has shown that communities can change in MFCs over a period of several months [63]. Furthermore, it is well understood that start up conditions can significantly affect the performance of MECs in the long term [64]. Adaptation to new substrates used on mature anodic biofilms is analogous to other kinds of startup conditions and, therefore, requires more study using different time scales. Further study may not only include the time of adaptation, but also the sequence of adaptation. Only one sequence of substrates was used in this study, and other sequences could yield different results.

Finally, a much deeper investigation of the structural and functional dynamics of the microbial community can be useful to fully examine the community interactions. This may best be done by analyzing gene expression observed in the community, which requires sophisticated omics techniques to reveal functional phenotypes of complimentary community members. Not requiring a specific community composition to achieve performance targets could be a boon for advancing MEC technology. If functional similarities across different communities can be realized while starting with a diverse microbial community, MEC design and process conditions could be sufficient to achieve target performance.

## Conclusions

MECs demonstrated here could convert a wide variety of substrates, including complex feedstocks, yielding high performance with a 1-week adaptation period. The results suggest that MECs can rapidly transition from one complex feedstock to another without the need for new inoculation or mechanical disruption of the biofilm. 16S rRNA gene sequencing showed that community structure was not correlated to performance, however, transitioning between substrates changed the communities. Differences were observed in the replicates with respect to the community structure, indicating that specific composition of community is not necessary for the substrates examined to achieve a target high performance. Use of acetate could be correlated with performance, however, effective conversion of complex substrates in MECs was not found to be strongly correlated with any one specific compound. Fermentable compounds were removed by microbial action with or without coupling to exoelectrogenesis, as observed via open circuit experiments, which showed accumulation of intermediates. Some compounds like phenol were observed to be sorbed in MECs, leading to abiotic removal. PCA showed that electrochemical performance metrics of MECs were more tightly correlated with substrate than the community structure. Further understanding of the behavior can come from more rigorous methodological experiments that expand on one or more of the conditions tested.

## Methods

### MEC construction and operation

MECs were constructed using two chamber design elements described previously [22]. Two duplicate MECs were used in the experiments. Briefly, 3.81 cm (1.5 in) inner diameter PVC pipe was cut to a 1.27 cm (0.5 in) length ring and was used for both anode and cathode chambers. The rings were pressed against polycarbonate plates and screwed tight to form a gas-tight seal. The anode was made of steam sterilized carbon felt prior to inoculation. A 0.318 cm (0.125 in) stainless steel rod was used as the current collector and came in direct contact with the carbon felt. A Nafion 115 membrane separated the two chambers. Pt deposited carbon with a loading of 0.5 mg/cm^2^ was used as the cathode catalyst, contacting a stainless-steel mesh for current collection. Cathode gas was collected using Viton hosing exiting the chamber into a 250 ml inverted graduated cylinder. Anode liquid medium included trace vitamins and minerals originally described by Wolin et al. [65], more commonly known as Wolfe’s mineral and vitamin solutions. In addition, the medium included 5.8 mM NH_4_Cl and 1.7 mM KCl, as well as buffered with 53.1 mM phosphate using a combination of NaH_2_PO_4_ and Na_2_HPO_4_ adjusted to a pH of 7.25. Anode liquid medium was replaced before every experiment. The medium was recycled and flown through the anode using a peristaltic pump at a flow rate of 3.5 ml/min, where the total anode liquid volume was 180 ml. Prior to experiments, cathodes were drained of accumulated catholyte and rinsed with deionized anaerobic water. No cathode buffer was used in any of the MECs, and accumulated catholyte was recycled back into the anode liquid medium every 24 h.

A VSP BioLogic potentiostat (BioLogic, Knoxville, TN) was used to poise the anodes of the MECs, which were operated as a three electrode assembly. Anode voltages were poised using an Ag/AgCl reference electrode that was inserted into the anode chamber, contacting the anode liquid medium and placed close to the anode. MEC anodes were poised at − 0.2 V versus the Ag/AgCl reference electrode. Reference electrodes were changed bi-monthly to maintain proper poising potential. Whole cell voltage was recorded using a DATAQ (DATAQ Instruments, Akron, Ohio) DI-1100, which recorded the whole cell voltage every minute. WinDAQ software provided by DATAQ was run on a Microsoft Windows operated computer, which was used to record the voltage.

MECs were first inoculated with a starting felt sample from an MEC community that had been previously operated on BOAP described previously [24]. Felt inoculum was inserted in the anode chamber as a 0.635 cm (0.25 in) diameter sample. Two samples from the previously described BOAP-fed MECs were inserted into the sterile felt in the new reactors using a flame sterilized coring bit to remove felt from the mature anode felt. The coring bit was also used to cut sterile felt for replacement. Inoculation was first conducted by opening the MECs in an anaerobic chamber. Additional inoculum from other operating reactors was supplemented to MECs before experiments by injecting anode nutrient medium from other reactors into the entry port of the anode. The reactors were fed a batch of 0.2 g/L glucose and acetate, and then 2 g/L-day BOAP. The organic loading was increased by an additional 2 g/L-day every 24 h until current no longer increased, or the organic loading rate reached 10 g/L-day, representing a carbon ramp. Once current plateaued, anode liquid medium was changed, and the ramp in organic loading was started again. Acetate and glucose were added in batches of 0.2 g/L sparingly to both reactors when current stalled but was stopped once MECs reached 10 g/L-day.

Following MEC maturation, experiments were performed using 10 grams of chemical oxygen demand (COD) per liter of anode volume per day (g/L-day). The first substrate tested was BOAP, followed by ROBOAP, CFP, phe/ace, and then acetate, in that order. During closed circuit experiments, MECs were run for 72 h. 5 ml of sample was collected every 24 h for analysis. Following the 10 g/L-day closed circuit experiments, MEC anode liquid medium was replaced, and was tested for compound accumulation by operating the reactors in open circuit conditions for 8 h at 10 g/L-day. Closed circuit conditions were then applied for the remaining 40 h for a total experiment time of 48 h. MECs were transitioned to the next substrate using the carbon ramp used initially with BOAP. MECs fed with 10 g/L-day phe/ace feeding were tested under an additional condition, where 2.3 g/L of phenol was added to the anode liquid medium before open circuit/closed circuit experiments started. This concentration was shown to inhibit current production by 36% in MECs reported previously [17]. After 2.3 g/L phenol batch experiments, MECs were regrown using BOAP, and then transitioned to acetate using the carbon ramp described earlier in this section.

### MEC electrochemical analysis

Electrochemical analysis was performed as previously described [22, 66]. Briefly, the productivity metrics calculated included average current density, in units of Amps per meter squared of projected surface area (A/m^2^), Hydrogen productivity in units of liters of H_2_ per liter of anode volume per day (L/L-day). Efficiency metrics included anode Coulombic efficiency, cathode conversion efficiency, the multiplication of these efficiency metrics to calculate hydrogen recovery, electrical efficiency, and overall energy efficiency. Hydrogen production rates were adjusted based on measurements from Gas Chromatography. Gas Chromatography (GC) was performed using a Thermo Focus GC (Thermo Fisher Scientific, Waltham, MA) using a method described previously [22]. Samples were run on the instrument for 8 min using a HP-PLOT (Agilent Technologies, Santa Clara, CA) Molesieve 5A column. For continuous addition experiments, these metrics were calculated every 24 h and cumulatively. For open circuit experiments, these metrics were calculated for the first 8 h, the next 16 h during closed circuit conditions, and then the following 24 h, as well as cumulatively. For closed-circuit experiments, an electron balance was conducted using the gas accumulated in the cathode and anode, and the gas makeup determined by GC. Henry’s law was used to estimate the amount of methane present in the anode liquid media, while the anode headspace was estimated to be between 182 and 185 mL. Each mole of methane corresponds to 8 moles of electron equivalents. Once scaled to electron equivalents, this value was divided by the electron equivalent moles of COD degraded in MECs to find the methane efficiency. Methane efficiency was calculated along with Coulombic efficiency, and the difference between the sum of these efficiencies and 100% was used to find the mole fraction of electrons that contributed to undefined sinks.

### Compound detection in MECs

HPLC was used to quantify compounds of interest using methods that have been previously described [66]. Briefly, two detectors and one column were used to detect compounds. A Shimadzu (Shimadzu, Torrance, CA) photo diode array (PDA) and a refractive index detector (RID) were used to detect compounds. The model for the PDA was an SPD-M20A, and the model number for the RID was an RID-20A. Both detectors were operated at 50 °C. The RID was only operated under a single detection wavelength, and the PDA was operated to detect the maximum value between 190 and 330 nm to represent the chromatograph. A Bio-Rad (Bio-Rad, Hercules, CA) Animex-87H was used as the HPLC column and was operated at 60 °C for all samples and standards. 5 mM H_2_SO_4_ was used as the mobile phase, which was pumped at a flow rate of 0.5 ml/min for all samples. A 10% isopropyl rinse was used with every sample and a 5 mM H_2_SO_4_ acid blank was used every 5–6 samples to keep the instrument lines clean. Compound standards were prepared for each fresh batch of mobile phase at three different concentrations.

Compounds of interested were influenced from prior work [22, 66]. Compound classes included organic acids, phenolics, and furans. The PDA was used to identify furans and phenolics, while the RID was used to identify organic acids. Organic acids included acetic acid, propionic acid, and lactic acid. Phenolics included phenol, catechol, vanillic acid, and 4-hydroxybenzaldehyde. Furans included furfural and 5-hydroxymethylfurfural. Substrates were tested along with standard concentrations of compounds to determined starting and ending concentration of compounds. Removal was determined by calculating the amount added by syringe pump and recording the difference in concentration from what was expected to what was found in the anode media.

During closed circuit conditions, acetate gains and losses did not contribute to electricity production, while acetate removal during closed circuit conditions would have contributed to current production. To account for the removal of acetate more accurately, either as it is delivered or created by biological transformation, a “net acetic acid removal rate” metric was established that took the difference between the rates of acetic acid removal observed during closed circuit and open circuit conditions.

### Microbial community characterization

After the 10 g/L-day experiment for each substrate, an anode felt sample was extracted from each reactor, and was replaced with a sterile felt piece for the open circuit experiments. The faceplate on the anode had a hole and a rubber stopper press fit using a bracket and stainless-steel screws to allow easy access to the felt for sampling. When sampling occurred, the faceplate and rubber stoppers were removed in an anaerobic chamber. Felt samples for sequencing were stored at − 75 °C before DNA extraction. DNA extraction was performed using a QIAGEN (QIAGEN, Hilden, Germany) Powersoil Pro kit, following the procedure described with the kit. Prior to use in the kit, felt was thawed and cut into small pieces using a flame and ethanol sterilized blade before cell lysis. Quality assurance and quality control (QA/QC) was then performed in line with previous studies [67–69]. The DNA was initially quantified and confirmed for sufficiency using a Nanodrop (ThermoFisher Scientific, Waltham, MA) Spectrophotemer. Once DNA was confirmed to be successfully extracted, Polymerase Chain Reaction (PCR) was then performed on 1–10 μL of extracted DNA. Amplicons were then initially visualized using a 1% agarose electrophoresis gel, which was operated at 60 V for 60 min. This was followed by analyzing the amplicons using an Agilent Bioanalyzer (Agilent technologies Santa Clara, CA). Amplicons were then quantified using a Qubit fluorometer (ThermoFisher Scientific, Waltham, MA), and were further quantified using a NEBNext Library Quant Kit for Illumina (New England Biolabs, Ipswich, MA), following the protocol outlined by the manufacturers. DNA was then sequenced using a MiSeq V2 kit and run on an Illumina MiSeq (Illunima, San Diego, CA). Digitized sequence data from the MiSeq was processed using QIIME2 [70] installed on a Linux Server. Data was denoised using DADA2 [71] available in QIIME2. Genus level identification of sequences was determined using the Silva database [72–74]. Operational taxonomical units (OTUs) were determined from the database, and sample populations were normalized by total sequence count to find the relative abundance of each OTU. Sample alpha diversity was calculated by rarifying the OTU tables using the minimum OTU count across the samples, and three alpha diversity indices were calculated: Chao1, Shannon, and Simpson.

### Statistical analysis

Linear regression was used to identify the correlation between current density and key organic acid removal rates, including acetic and propionic acid. Pearson correlations were also calculated to compare the average current density of the MECs with alpha diversity metrics. PCA was used to track the similarity between variables documented, including relative OTU abundance of key microbes, current density, hydrogen productivity, COD removal, and net acetic acid removal. PCA was conducted using SPSS software (IBM Corporation, Armonk, NY), and was rotated using Oblimin rotation.

## Supplementary information

**Additional file 1: Figure S1.** Correlation between current density and hydrogen productivity across all substrates tested. **Figure S2**. Compound accumulation and removal percentage of MECs fed 10 g/L-day of each substrate. Substrates include BOAP, red oak BOAP (ROBOAP), corn stover fermentation product (CFP), acetate (ACE), and equal fractions by COD of phenol and acetate (PHE/ACE). Compounds include acetic acid (AA), furfural (FF), 5-hydroxymethylfurfural (HMF), vanillic acid (VA), catechol (CAT), phenol (PHE), propionic acid (PA), and lactic acid (LA). **Figure S3.** Compound accumulation and removal percentage during open circuit conditions (8 hr) and during closed circuit conditions at 24 hour and 48 hour time points. Compounds include acetic acid (AA), furfural (FF), 5-hydroxymethylfurfural (HMF), vanillic acid (VA), catechol (CAT), phenol (PHE), propionic acid (PA), and lactic acid (LA). Substrates include switchgrass BOAP (BOAP), red oak BOAP (ROBOAP), corn stover fermentation product (CFP), acetate (ACE), and equal fractions by COD of phenol and acetate (PHE/ACE). **Figure S3**. (D) also includes the conditions, where 2.3 g/L of phenol were added, labeled as a batch. **Table S1.** pearson correlation coefficients between current density and relative abundance of select OTUs in individual replicates and among both data sets. **Table S2**. diversity indices calculated from rarified 16S rRNA data. Pearson correlation coefficients between diversity indices and current density were low.

## Data Availability

Not applicable.

## Abbreviations

BES: Bioelectrochemical system
BOAP: Switchgrass bio-oil aqueous phase
CFP: Corn stover fermentation product
GC: Gas chromatography
HPLC: High-performance liquid chromatography
MEC: Microbial electrolysis cell
MFC: Microbial fuel cell
OTU: Operational taxonomical unit
RID: Refractive index detector
ROBOAP: Red oak bio-oil aqueous phase
PDA: Photo diode array
PCA: Principal component analysis
phe/ace: Phenol acetate blend substrate
VFA: Volatile fatty acid
