# Supplementary material for: Performance and community structure dynamics of microbial electrolysis cells operated on multiple complex feedstocks
Source: Biotechnol Biofuels. 2020 Oct 13;13:169. doi: 10.1186/s13068-020-01803-y (PMC7552531; doi:10.1186/s13068-020-01803-y)
Supplement: Supplementary file 1 — Additional file 1: Figure S1. Correlation between current density and hydrogen productivity across all substrates tested. Figure S2. Compound accumulation and removal percentage of MECs fed 10 g/L-day of each substrate. Substrates include BOAP, red oak BOAP (ROBOAP), corn stover fermentation product (CFP), acetate (ACE), and equal fractions by COD of phenol and acetate (PHE/ACE). Compounds include acetic acid (AA), furfural (FF), 5-hydroxymethylfurfural (HMF), vanillic acid (VA), catechol (CAT), phenol (PHE), propionic acid (PA), and lactic acid (LA). Figure S3. Compound accumulation and removal percentage during open circuit conditions (8 hr) and during closed circuit conditions at 24 hour and 48 hour time points. Compounds include acetic acid (AA), furfural (FF), 5-hydroxymethylfurfural (HMF), vanillic acid (VA), catechol (CAT), phenol (PHE), propionic acid (PA), and lactic acid (LA). Substrates include switchgrass BOAP (BOAP), red oak BOAP (ROBOAP), corn stover fermentation product (CFP), acetate (ACE), and equal fractions by COD of phenol and acetate (PHE/ACE). Figure S3. (D) also includes the conditions, where 2.3 g/L of phenol were added, labeled as a batch. Table S1. pearson correlation coefficients between current density and relative abundance of select OTUs in individual replicates and among both data sets. Table S2. diversity indices calculated from rarified 16S rRNA data. Pearson correlation coefficients between diversity indices and current density were low. [file 13068_2020_1803_MOESM1_ESM.docx]

# Additional file 1

Figure S1: Correlation between current density and hydrogen productivity across all substrates tested.

| (A) BOAP | |
| --- | --- |
| (B) ROBOAP | |
| (C) CFP | |
| (D) PHE/ACE | (E) ACE |

*Figure S2: Compound accumulation and removal percentage of MECs fed 10 g/L-day of each substrate. Substrates include BOAP, red oak BOAP (ROBOAP), corn stover fermentation product (CFP), acetate (ACE), and equal fractions by COD of phenol and acetate (PHE/ACE). Compounds include acetic acid (AA), furfural (FF), 5-hydroxymethylfurfural (HMF), vanillic acid (VA), catechol (CAT), phenol (PHE), propionic acid (PA), and lactic acid (LA).*

| (A) BOAP | |
| --- | --- |
| (B) ROBOAP | |
| (C) CFP | |
| (D) PHE/ACE | (E) ACE |

Figure S3: Compound accumulation and removal percentage during open circuit conditions (8 hr) and during closed circuit conditions at 24 hour and 48 hour time points. Compounds include acetic acid (AA), furfural (FF), 5-hydroxymethylfurfural (HMF), vanillic acid (VA), catechol (CAT), phenol (PHE), propionic acid (PA), and lactic acid (LA). Substrates include BOAP, red oak BOAP (ROBOAP), corn stover fermentation product (CFP), acetate (ACE), and equal fractions by COD of phenol and acetate (PHE/ACE). Figure S3(D) also includes the conditions where 2.3 g/L of phenol were added, labeled as a batch.

| **OTU** | **R**  **(Replicate A)** | **R**  **(Replicate B)** | **R**  **(Replicate A & B)** |
| --- | --- | --- | --- |
| ***Geobacter*** | 0.28 | 0.82 | 0.45 |
| ***Robinsoniella*** | -0.50 | -0.25 | -0.33 |
| ***Paludibacteraceae - unknown*** | 0.90 | -0.32 | -0.02 |
| ***Methanobrevibacter*** | 0.65 | -0.49 | 0.28 |
| ***Sphaerochaeta*** | 0.23 | -0.51 | -0.30 |
| ***Desulfovibrio*** | -0.16 | -0.41 | -0.23 |
| ***Anaerocella*** | 0.00 | -0.54 | -0.35 |
| ***Eubacterium*** | -0.47 | -0.58 | -0.22 |
| ***Erysipelotrichaceae UCG-004*** | 0.50 | 0.07 | 0.36 |
| ***Eubacteriaceae - unknown*** | -0.25 | -0.36 | -0.24 |
| ***Candidatus Methanoplasma*** | -0.28 | -0.30 | -0.29 |
| **other** | 0.35 | -0.84 | -0.16 |

Table S1: pearson correlation coefficients between current density and relative abundance of select OTUs in individual replicates and among both data sets.

| **Replicate** | **Substrate** | **Chao1** | **Simpson** | **Shannon** |
| --- | --- | --- | --- | --- |
| **A** | **BOAP** | 87.0 | 0.817 | 3.78 |
| **A** | **ROBOAP** | 79.0 | 0.889 | 4.14 |
| **A** | **CFP** | 71.0 | 0.915 | 4.61 |
| **A** | **PHE/ACE** | 91.8 | 0.914 | 4.25 |
| **A** | **ACE** | 102.4 | 0.899 | 4.37 |
| **B** | **BOAP** | 79.0 | 0.802 | 3.84 |
| **B** | **ROBOAP** | 87.7 | 0.906 | 4.16 |
| **B** | **CFP** | 112.4 | 0.889 | 4.23 |
| **B** | **PHE/ACE** | 87.0 | 0.913 | 4.38 |
| **B** | **ACE** | 44.0 | 0.383 | 1.58 |

Table S2: diversity indices calculated from rarified 16S rRNA data. Pearson correlation coefficients between diversity indices and current density were low.
